# Mapping Interindividual Variation in the Aging Connectome Study Results: Examining Fitness and Executive Control

**DOI:** 10.1093/geroni/igaf122.4160

**Published:** 2025-12-31

**Authors:** Stan Colcombe, Anna MacKay-Brandt

**Affiliations:** Nathan Kline Institute, Orangeburg, New York, United States; The Nathan S. Kline Institute for Psychiatric Research, Orangeburg, New York, United States

## Abstract

Translational geroscience seeks to identify modifiable factors that can slow or prevent age-related functional decline. Cardiovascular fitness (CF) is one such factor, with intervention studies showing that fitness improvements enhance brain plasticity and executive control. However, little is known about normative variation in CF across mid- to late adulthood or how it predicts cognitive change under naturalistic conditions. We examined data from Mapping Interindividual Variation in the Aging Connectome, a community-ascertained sample aged 40–74 years. Participants completed annual assessments of CF (VO_2_submax) and executive control (Attention Network Task [ANT] conflict effect) over three years. Among individuals with three assessments (n = 155) fitness was highly stable, ICC(2,1) = 0.83. Such high absolute agreement over time was contrary to our expectation of intraindividual variation, and possibly indicative of stable VO2 set points. However, ANT conflict effect was less stable, ICC(2,1) = 0.57, and decreased over time, consistent with practice effects. Notably, higher VO_2_max at baseline predicted greater practice effects at follow-up (β = –1.06, p = 0.030, R^2^ = 0.022). Although intensive programs can improve CF and executive control, our findings suggest that under naturalistic conditions CF is a stable physiological trait in later adulthood. Remarkably, the plasticity of the system at baseline, indexed by CF, appears to result in greater cognitive gains 1 to 2 years later - underscoring the utility of VO2 as a biomarker of brain plasticity. The stability of VO2 around individual set points suggests caution in interpreting exercise studies that lack gold standard VO2 assessment.

